# P51 Neuropsychiatric lupus, two sides of the same coin

**DOI:** 10.1093/rap/rkae117.082

**Published:** 2024-11-05

**Authors:** Lilase Hakoun, Sandhya Govindarajan, Sajida Somiya Rasul, Alice Chieng

**Affiliations:** Royal Manchester Children’s Hospital, Manchester, United Kingdom; Royal Manchester Children’s Hospital, Manchester, United Kingdom; Royal Manchester Children’s Hospital, Manchester, United Kingdom; Royal Manchester Children’s Hospital, Manchester, United Kingdom

## Abstract

**Introduction:**

Neuropsychiatric lupus, a complex aspect of systemic lupus erythematosus, lacks a consistent pattern, and its classification criteria serve for research purposes which may provide an aid when assessing features but are not a formal diagnostic tool. This report describes two unique cases: one with psychiatric illness and safeguarding concerns, the other with a worsening movement disorder. Both cases were challenging due to the absence of obvious lupus features initially. Frequent assessments and a multidisciplinary collaborative approach ultimately led to favourable outcomes for both patients as they responded to standard lupus management regime.

**Case description:**

Patient A, a 16-year-old female, presented acutely with psychosis after months of cognitive decline, low mood, hair loss, and fatigue. She exhibited attempts to abscond, poor sleep, aggression, confusion, agitation, hypomania, and catatonia. Neurological signs included increased lower limb tone, absent reflexes, abnormal posturing, and speech disturbances. Teams involved in her management included psychiatry, paediatric neurology, metabolic and rheumatology. She was treated for neuropsychiatric lupus due to positive anti-nuclear, SS-A, and SS-A52 antibodies, psychosis, academic decline, and brain imaging showing loss of volume and corpus callosum thinning. Conversely, there were no mucocutaneous, joint, headache, chest, or abdominal features. She had normal CRP and ESR, and consistently negative double-stranded DNA. The treatment strategy meticulously balanced steroid administration to prevent exacerbation of her psychiatric condition.

Patient B, a 13-year-old female, initially presented with abnormal facial and hand sensations, describing her ring and index fingers as locked, slurred speech, and involuntary right-sided movements. Initially diagnosed with Sydenham’s chorea following a sore throat and fever, her symptoms improved with physiotherapy. However, a year later, her movement disorder recurred, accompanied by loss of balance resulting in a fall and fractures, blurred vision, nystagmus with vertigo, intermittent headaches, and hemisensory facial disturbance, significantly affecting her quality of life. Initial brain imaging showed non-specific foci, later identified as small inflammatory foci, which resolved but subsequent imaging revealed white matter hyperintensities suggesting a neuroinflammatory disorder. Spinal imaging was normal. Elevated ESR despite low CRP, low C3 levels, positive ANA, anti-TPO, lupus anticoagulant, beta 2 glycoprotein antibodies, and high chromatin levels on ENA. In both cases, the dsDNA was negative. Some of the haematological and immunological features were suggestive of lupus, scored using SLICC and ACR EULAR guidance.

**Discussion:**

Neuropsychiatric lupus, a rare and complex manifestation of systemic lupus erythematosus, poses significant diagnostic challenges due to its diverse and subtle presentations. Lack of gold standard investigations necessitate a comprehensive, multidisciplinary approach for accurate diagnosis. Differential diagnoses must exclude infections, metabolic disorders, drug reactions, and other illnesses. Neurological assessments focus on headaches, seizure signs, alertness, and motor and sensory deficits, while psychiatric evaluations cover behaviour, cognition, perception, mood, and affect.

Patient A exhibited acute psychiatric symptoms followed by neurological signs, while Patient B showed subtler movement disorder symptoms. Both had brain changes on magnetic resonance imaging, rendering it more difficult. Brain atrophy however, is a recognized finding in lupus. These cases highlight the importance of maintaining an open mind, re-visiting pre-established diagnoses by reassess clinical state of patient with the aid of serum investigations, radiological findings and expert opinion. Expertise from tertiary subspecialties facilitated a unified diagnosis of lupus. Safeguarding measures included assessing mental capacity and obtaining guardian consent for treatment.

These cases emphasize the importance of a vigilant, multidisciplinary approach in diagnosing and managing neuropsychiatric lupus, particularly in paediatric cases where symptoms may be atypical. Collaborative input from physiotherapists, occupational therapists, psychologists, and dieticians ensures a holistic, patient-centred approach, crucial for improving outcomes.

The management regimen for the patients required understanding the disease activity in the absence of pure neuropsychiatric lupus scoring system. Treatment provided included intravenous pulsed methylprednisolone followed by oral prednisolone, hydroxychloroquine, cyclophosphamide, and rituximab. This therapeutic approach elicited a favourable initial response in both cases.

**Key learning points:**

• Broad approach to a seemingly obvious presentation. Diagnostic challenges require a comprehensive evaluation, revisiting and questioning pre-established concepts.

• Multidisciplinary team collaboration involving various medical specialties enhances diagnostic accuracy and treatment efficacy.

• Utilization of classification criteria guides investigation but does not solely determine diagnosis due to the complexity of presentations. An evidence-based approach necessitate careful weighing of established criteria in the context of each individual presentation.

• Continuous management and monitoring of disease activity are essential due to the absence of typical lupus features, allowing for the consideration of evolving lupus.

• Despite varied clinical presentations, both patients underwent a consistent treatment regimen resulting in positive outcomes.

• Comprehensive assessment of mental capacity and obtaining guardian consent, especially critical in paediatric cases.

• Achieving disease control is following by ongoing assessment of disease activity, aiming for remission.

• Transparent communication with patients and their families, involving them in the process improves outcome.

• Focus on quality of life, continuous assessment of fatigue, level of activity, anxiety, mood is an important aspect of management.

